# Natural History and Outcomes of Patients With Moderate and Moderate-Severe Mitral Regurgitation

**DOI:** 10.1016/j.jacadv.2026.103057

**Published:** 2026-07-23

**Authors:** Abdullah Al-Abcha, Ghasaq Saleh, Salameh Al-Halaseh, Jwan Naser, Trevor Simard, Jeremy Thaden, Sorin Pislaru, Mackram Eleid, Mayra Guerrero, Mohamad Alkhouli, Charanjit S. Rihal, Ratnasari Padang, Benjamin Hibbert

**Affiliations:** Department of Cardiovascular Medicine, Mayo Clinic, Rochester, Minnesota, USA

**Keywords:** death, degenerative MR, functional MR, heart failure, mitral regurgitation, mitral valve intervention

## Abstract

**Background:**

The natural history of mitral regurgitation (MR) in contemporary practice remains poorly defined.

**Objectives:**

The purpose of this study was to quantify clinical outcomes of patients with moderate and moderate-severe MR.

**Methods:**

We conducted a retrospective cohort study of consecutive patients with moderate or moderate-severe MR who underwent echocardiography at Mayo Clinic between 2010 and 2023. MR was classified as degenerative or functional. The primary outcome was all-cause mortality. Secondary outcomes included heart failure (HF) hospitalization, MR progression to severe MR, and mitral valve intervention.

**Results:**

Of 8,687 patients (mean age 70.7 ± 13.7 years; 45.1% female), 21.4% had degenerative MR and 78.6% had functional MR, and median follow-up was 4.1 years (Q1-Q3: 2.3-7.9). At 4 years, overall survival was 68.5% (95% CI: 67.4%-69.7%), with higher survival in patients with degenerative MR (84.1%; 95% CI: 81.9%-86.3%) compared to functional MR (65.0%; 95% CI: 63.7%-66.3%), and the cumulative incidence of HF hospitalization was higher in functional MR (36.8%; 95% CI: 35.6%-38.1%) than degenerative MR (17.9%; 95% CI: 15.7%-20%). However, the risk of death and HF hospitalization was similar after multivariable adjustments. MR progression occurred in 32.7% (95% CI: 31.0%-34.4%), less commonly in functional MR (adjusted HR: 0.65; 95% CI: 0.54-0.79; *P* < 0.001). Mitral valve intervention was infrequent (11.4%; 95% CI: 10.6%-12.1%) and significantly less common in functional MR (adjusted HR: 0.45; 95% CI: 0.37-0.56; *P* < 0.001).

**Conclusions:**

Patients with moderate or moderate-severe MR accrue significant mortality and morbidity over follow-up. Further trials are needed to ascertain the indication, optimal timing and method of procedural intervention.

Mitral regurgitation (MR) is the second most common valvular lesion, with rising prevalence in cohorts with established heart disease.^1^, ^2^, ^3^ Etiology of MR can be broadly categorized into degenerative and functional MR. Degenerative MR is related to leaflet degeneration, while functional MR is predominantly related to incomplete valve coaptation from left atrial or ventricular disease/remodeling.^4^ Regardless of the etiology, severe MR is known to be associated with increased risk of adverse events, including heart failure (HF) hospitalization and all-cause mortality, with guidelines recommending a class I indication for mitral valve (MV) interventions in all symptomatic patients with severe MR.^5^, ^6^, ^7^

Moderate MR has garnered increased recognition, with randomized trials in severe MR suggesting a benefit of transcatheter edge-to-edge repair, though evidence specific to moderate MR remains limited.^8^^,^^9^ Traditionally, management was a conservative strategy including medical optimization and surveillance in this cohort of patients. However, there is limited contemporary evidence of the clinical event rates in those with moderate MR. Herein, we report the natural history of patients with moderate MR, including those with moderate-severe MR, in a contemporary cohort of patients.

## Methods

### Study population

We conducted a retrospective cohort study of patients who underwent transthoracic echocardiography (TTE) at Mayo Clinic Rochester between January 1, 2010, and December 31, 2023, and were diagnosed with moderate or moderate-severe MR. Institutional Review Board approval was obtained for the study, which was conducted in accordance with the principles outlined in the Declaration of Helsinki, and only patients providing authorization for the use of the medical records were included. The study followed the Strengthening the Reporting of Observational Studies in Epidemiology reporting guideline.

Patients were eligible for inclusion if they had moderate or moderate-severe MR, available echocardiographic and clinical data at the time of index evaluation and had at least clinical data during follow-up. We excluded individuals with prior MV or any valvular intervention, any degree of mitral stenosis, severe mitral annular calcification, those who underwent MV intervention within 30 days of index TTE, those with moderate or greater aortic valve disease, congenital heart disease, radiation heart disease, carcinoid heart disease, rheumatic heart disease, amyloidosis, hemochromatosis, hypertrophic cardiomyopathy, sarcoidosis, history of or active infective endocarditis, or incomplete data required for MR quantification or phenotyping. All echocardiographic parameters were measured according to American Society of Echocardiography guidelines.^10^ In our study, coronary artery disease was defined as a history of myocardial infarction, percutaneous coronary intervention, coronary artery bypass grafting, or angiographically confirmed coronary artery disease with at least 50% stenosis in one or more epicardial vessels.

### MR definition

MR etiology was classified using a protocolized framework incorporating anatomical and functional features derived from the index TTE. Degenerative MR was defined by the presence of MV prolapse, while functional MR included all other patients. Functional MR was further subdivided into atrial functional MR (AFMR), which was defined as MR with preserved left ventricular ejection fraction (LVEF ≥50%) and presence of left atrial enlargement (left atrial volume index [LAVI] ≥40 mL/m^2^). Ventricular functional MR (VFMR) was defined in the absence of AFMR, with either LVEF <50% and more than mild left ventricular enlargement or the presence of regional wall motion abnormalities. Patients who did not meet any of these criteria were labeled as “mixed” FMR.

### Outcomes

The primary study outcome was all-cause mortality. Secondary outcome measures were HF hospitalization, procedural MV intervention, and progression to severe MR. The mortality endpoint was defined as time to death from baseline for all deceased patients and time to censor date (last known follow-up) from baseline among the remainder. Data on HF hospitalizations and MV intervention were obtained from electronic health record systems. Data on progression to severe MR were obtained from TTE and transesophageal echocardiograms at follow-up. To assess the natural history and survival rates of included patients, patients who underwent any MV interventions were censored at the time of intervention.

### Secondary analysis

To compare clinical outcomes across the phenotypes of FMR, we performed subgroup analysis in patients with AFMR vs VFMR. We investigated all-cause mortality and HF hospitalization at follow-up in each subgroup.

### Statistical analysis

Continuous variables are reported as mean (SD) or median (IQR) as appropriate, and categorical variables as frequency (percentage). Comparisons between groups were performed using the Wilcoxon rank sum test or Student’s *t*-test for continuous variables and the chi-square test or Fisher exact test for categorical variables, as appropriate. The Kaplan-Meier method was used for all-cause mortality, whereas competing risk analysis was used to estimate the cumulative incidence of HF hospitalization, MV intervention, and progression to severe MR accounting for the competing risk of death. In these analyses, patients were censored at the time of MV intervention or the last available follow-up for those without an event or intervention. As a sensitivity analysis, MV intervention was treated as a competing event for all-cause mortality using the cause-specific cumulative incidence framework. Multivariable Cox proportional hazards models were used to assess predictors of all-cause mortality. The proportional hazards assumption was tested using Schoenfeld residuals. The Fine-Gray subdistribution hazard model was used for the competing risk analyses to account for the nonindependence of death as a competing event. Variables in the multivariable models were selected on a priori basis including age, sex, body mass index, diabetes mellitus (DM), lung disease, chronic kidney disease, coronary artery disease, hypertension, stroke, history of congestive HF, atrial fibrillation, functional MR, moderate-severe MR, LVEF, LAVI, mitral annular calcification, medial E/é, right ventricular systolic pressure (RVSP), moderate or severe tricuspid regurgitation (TR), left ventricular hypertrophy, and right ventricular dilation. All analyses were performed in R version 4.4.0 and BlueSky Statistics version 10.3.4. A 2-sided *P* value <0.05 was considered statistically significant.

## Results

A total of 8,687 patients with moderate or moderate-severe MR were included (Table 1). The mean age of the population was 70.7 ± 13.7 years, 45.1% were females, and the mean body mass index was 28.2 kg/m^2^. In terms of comorbidities, 62.1% had hypertension, 43.2% had coronary artery disease, and 38.5% had atrial fibrillation. There were 1,859 (21.4%) patients with degenerative MR and 6,828 (78.6%) with functional MR. Compared to those with degenerative MR, patients with functional MR were older and had a higher burden of baseline comorbidities including history of congestive HF, atrial fibrillation, coronary artery disease, hypertension, DM, and chronic kidney disease.

**Table 1:** Baseline Characteristics of the Included Population

	Degenerative MR (n = 1859)	Functional MR (n = 6,828)	Total (N = 8,687)	*P* Value
Age, y	67.2 ± 14.0	71.7 ± 13.5	70.7 ± 13.7	<0.001
Female	3,093 (45.3%)	822 (44.2%)	3,915 (45.1%)	0.406
Body mass index, kg/m^2^	25.6 ± 4.9	28.9 ± 6.3	28.2 ± 6.2	<0.001
Body surface area, m^2^	1.877 ± 0.2	1.941 ± 0.3	1.927 ± 0.3	<0.001
Hypertension	753 (40.5%)	4,641 (68.0%)	5,394 (62.1%)	<0.001
Diabetes mellitus	135 (7.3%)	2037 (29.8%)	2,172 (25.0%)	<0.001
Coronary artery disease	405 (21.8%)	3,352 (49.1%)	3,757 (43.2%)	<0.001
Percutaneous coronary intervention	54 (2.9%)	620 (9.1%)	674 (7.8%)	<0.001
Heart failure	205 (11.0%)	3,111 (45.6%)	3,316 (38.2%)	<0.001
Atrial fibrillation	368 (19.8%)	2,978 (43.6%)	3,346 (38.5%)	<0.001
Stroke	114 (6.1%)	871 (12.8%)	985 (11.3%)	<0.001
Lung disease	105 (5.6%)	721 (10.6%)	826 (9.5%)	<0.001
Obstructive sleep apnea	274 (14.7%)	1805 (26.4%)	2079 (23.9%)	<0.001
Renal disease	95 (5.1%)	1,247 (18.3%)	1,342 (15.4%)	<0.001
Nonskin cancer	319 (17.2%)	1,481 (21.7%)	1800 (20.7%)	<0.001

MR = mitral regurgitation.

Values are mean ± SD or n (%).

Baseline echocardiographic characteristics are listed in Table 2. In the total population, 74.9% had moderate MR, and 25.1% had moderate-severe MR. The mean effective regurgitant orifice area was 0.3 ± 0.1 cm^2^, and the mean regurgitant volume was 42.4 ± 10.0 mL. LVEF was 49% ± 15.7%, and 43.4% of the population had LVEF <50%. LAVI was 51.1 ± 18 mL/m^2^, RVSP was 41.6 ± 13.7 mmHg, and 41% had ≥ moderate TR. Compared to those with degenerative MR, patients with functional MR had a higher percentage of those with ≥moderate TR, lower LVEF, higher LAVI, and higher RVSP.

**Table 2:** Baseline Echocardiogram of the Total Population

	Degenerative MR (n = 1,859)	Functional MR (n = 6,828)	Total (N = 8,687)	*P* Value
Mitral regurgitation grade				<0.001
Moderate	1,249 (67.2%)	5,260 (77.0%)	6,509 (74.9%)	
Moderate-severe	610 (32.8%)	1,568 (23.0%)	2,178 (25.1%)	
Effective regurgitant orifice area, cm^2^	0.27 ± 0.08	0.25 ± 0.06	0.25 ± 0.07	<0.001
Regurgitant volume, ml	43.2 ± 11.2	42.2 ± 9.6	42.4 ± 10.0	<0.001
Mitral annular calcification	264 (14.2%)	2,118 (31.0%)	2,382 (27.4%)	<0.001
Ejection fraction, %	60.6 ± 7.3	45.9 ± 15.9	49.0 ± 15.7	<0.001
Ejection fraction ≤50%	141 (7.6%)	3,629 (53.1%)	3,770 (43.4%)	<0.001
LV end-systolic diameter, mm	33.4 ± 5.4	41.6 ± 12.0	39.8 ± 11.4	<0.001
LV end-diastolic diameter, mm	51.8 ± 5.8	55.0 ± 9.4	54.3 ± 8.8	<0.001
E wave	0.8 ± 0.2	1.0 ± 0.3	0.9 ± 0.3	<0.001
Medial E/é ratio	12.2 ± 5.2	18.6 ± 8.9	17.1 ± 8.7	<0.001
Left ventricular mass index	103.8 ± 24.1	121.2 ± 36.6	117.4 ± 35.0	<0.001
Left atrial volume index	47.4 ± 15.5	52.2 ± 18.5	51.1 ± 18.0	<0.001
TR velocity, m/sec	2.5 ± 0.4	2.9 ± 0.5	2.8 ± 0.5	<0.001
Right ventricular systolic pressure, mm Hg	32.4 ± 9.6	44.1 ± 13.5	41.6 ± 13.7	<0.001
Tricuspid regurgitation				<0.001
None/trivial	725 (39.7%)	1,306 (19.6%)	2031 (23.9%)	
Mild	704 (38.6%)	2,193 (32.9%)	2,897 (34.1%)	
Moderate	326 (17.9%)	2,282 (34.2%)	2,608 (30.7%)	
Severe	71 (3.9%)	885 (13.3%)	956 (11.3%)	
Right ventricular dilation	375 (20.2%)	2,873 (42.2%)	3,248 (37.5%)	<0.001
Right ventricular dysfunction	168 (9%)	2,812 (41.2%)	2,980 (34.3%)	<0.001

LV = left ventricular; TR = tricuspid regurgitation; other abbreviation as in Table 1.

### Clinical outcomes

At a median follow-up of 4.1 years (Q1-Q3: 2.3-7.9 years), there were 2,980 deaths. Survival estimates at 4 years were 68.5% (95% CI: 67.4%-69.7%) in the total population, 84.1% (95% CI: 81.9%-86.3%) in the degenerative MR group, and 65.0% (95% CI: 63.7%-66.3%) in the functional MR group, respectively (Central Illustration, Table 3). Unadjusted HR demonstrated a significantly higher rate of all-cause mortality in the functional MR group compared to the degenerative MR group (HR: 2.20; 95% CI: 1.96-2.47; *P* < 0.0001). In multivariable analysis, functional MR was no longer a predictor for all-cause mortality (HR: 1.09; 95% CI: 0.92-1.29; *P* = 0.347). Independent predictors of mortality included age, male sex, DM, renal disease, lower LVEF, higher LAVI, and RVSP (Supplemental Table 1). In a sensitivity analysis treating MV intervention as a competing event rather than censoring, results were consistent with the primary analysis (unadjusted HR: 2.20; 95% CI: 1.96-2.47; *P* < 0.0001; multivariable HR: 1.08; 95% CI: 0.92-1.28; *P* = 0.349), confirming our findings.

**Central Illustration:**
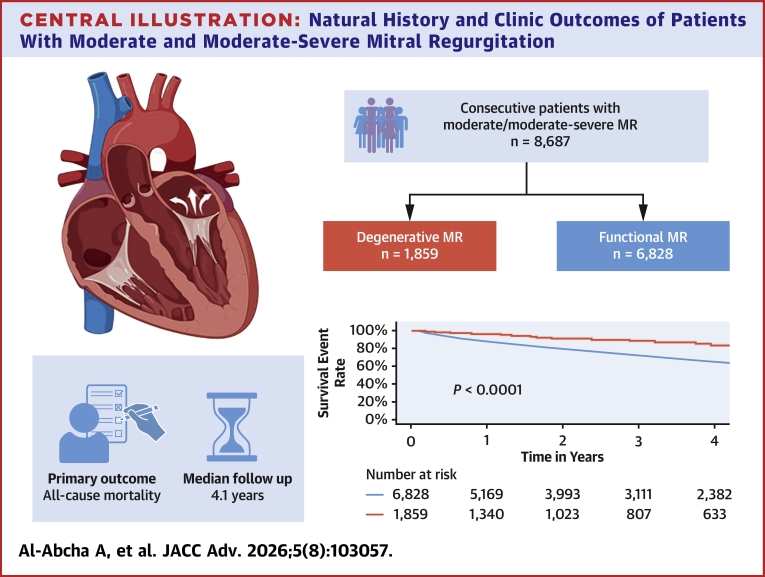
Natural History and Clinic Outcomes of Patients With Moderate and Moderate-Severe Mitral Regurgitation Abbreviation as in Figure 1.

**Table 3:** Survival and Heart Failure Hospitalization Estimates at Follow-Up

Clinical Outcomes	1 Year	4 Years
Survival		
Total population	89.6% (89.0%-90.3%)	68.5% (67.4%-69.7%)
Degenerative MR	96.3% (95.4%-97.2%)	84.0% (81.9%-86.3%)
Functional MR	88.0% (87.2%-88.8%)	65.0% (63.7%-66.3%)
VFMR	86.2% (84.9%-87.6%)	62.1% (60.0%-64.3%)
AFMR	90.7% (89.4%-92.1%)	68.1% (65.5%-70.7%)
Heart failure hospitalization		
Total population	18.5% (17.6%-19.3%)	33.1% (32.0%-34.2%)
Degenerative MR	7.0% (5.8%-8.3%)	17.7% (15.7%-20%)
Functional MR	21.4% (20.5%-22.4%)	36.8% (35.6%-38.1%)
VFMR	30.1% (28.5%-32.1%)	46.7% (44.6%-48.8%)
AFMR	15.1% (13.4%-16.8%)	31.0% (28.6%-33.5%)

AFMR = atrial functional mitral regurgitation; VFMR = ventricular functional mitral regurgitation; other abbreviation as in Table 1.

**Figure 1:**
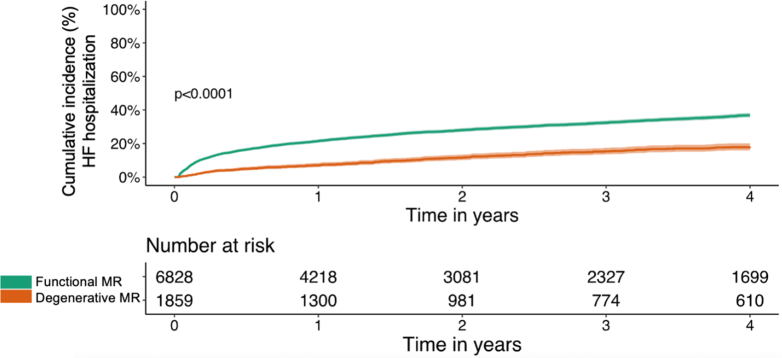
Cumulative Incidence of Heart Failure Hospitalization According to the Etiology of Mitral Regurgitation MR = mitral regurgitation; HF = heart failure.

In terms of HF hospitalization, the cumulative incidence of HF hospitalization at 4 years was 33.1% (95% CI: 32%-34.2%) in the total population, 17.7% (95% CI: 15.7%-20%) in the degenerative MR group and 36.8% (95% CI: 35.6%-38.1%) in the functional MR group (Figure 1). The incidence of HF hospitalization at 4 years was significantly higher in the functional MR group (HR: 2.04; 95% CI: 1.84-2.27; *P* < 0.001). In multivariable analysis, functional MR was not a predictor of HF hospitalization (HR: 1.03; 95% CI: 0.89-1.19; *P* = 0.703). Independent predictors of HF hospitalization included moderate-severe MR, body mass index, DM, renal disease, pulmonary disease, hypertension, history of HF, lower LVEF, medial E/e ratio, and RVSP (Supplemental Table 2).

**Figure 2:**
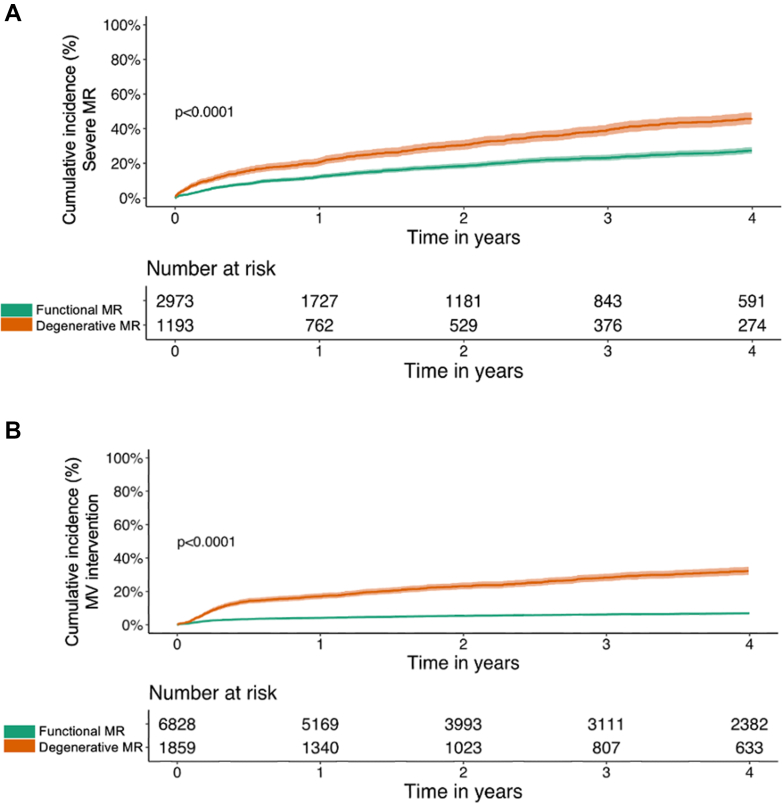
Secondary Outcomes Including Progression to Severe Mitral Regurgitation and Mitral Valve Intervention at Follow-Up (A) Cumulative incidence of severe mitral regurgitation according to the etiology of mitral regurgitation. (B) Cumulative incidence of mitral valve intervention according to the etiology of mitral regurgitation MV = mitral valve; other abbreviation as in Figure 1.

Follow-up echocardiogram data were available in 48% of patients (4,166/8,687). The cumulative incidence of progression to severe MR at 4-year follow-up was 32.7% (95% CI: 31.0%-34.4%) in the total population and it was significantly lower in the functional MR group (27.3%; 95% CI: 25.4%-29.2%) compared to the degenerative MR group (45.7%; 95% CI: 42.2%-49.1%; *P* < 0.0001) (Figure 2). In the multivariable Fine-Gray model, functional MR (HR: 0.65; 95% CI: 0.54-0.79; *P* < 0.001) was independent predictors of MR progression, indicating a significantly lower risk of progression compared to degenerative MR (Supplemental Table 3). Additionally, the cumulative incidence of MV intervention at 4 years was 11.4% (95% CI: 10.6%-12.1%) in the total population; 30.5% (95% CI: 28.2%-32.8%) in the degenerative MR group and 6.1% (95% CI: 5.5%-6.7%) in the functional MR group (Figure 2). The incidence of MV intervention at 4 years was significantly lower in the functional MR group (*P* < 0.001), and in multivariable analysis, functional MR was an independent predictor of MV intervention (HR: 0.45; 95% CI: 0.37-0.56; *P* < 0.0001), indicating a significantly lower risk of MV intervention compared to degenerative MR (Supplemental Table 4). Additionally, moderate-severe MR was a strong predictor of MR progression and MV intervention at follow-up.

### Subgroup analysis

**Figure 3:**
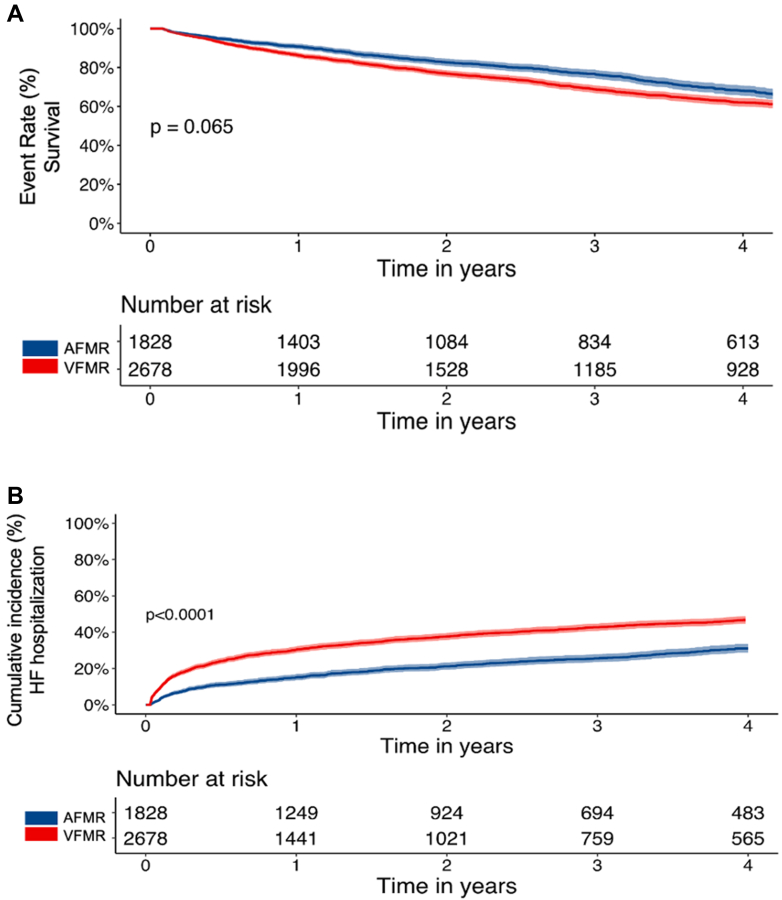
Clinical Outcomes of the Subgroup Analysis in the Functional Mitral Regurgitation Cohort (A) Kaplan-Meier curves of all-cause mortality according to the subgroups of functional mitral regurgitation. (B) Cumulative incidence of heart failure hospitalization according to the subgroups of functional mitral regurgitation AFMR = atrial functional mitral regurgitation; VFMR = ventricular functional mitral regurgitation; other abbreviation as in Figure 1.

Of the 8,687 patients included in this analysis, 2,678 (30.8%) had VFMR and 1,828 (21.1%) had AFMR. Baseline clinical and echocardiographic characteristics are reported in Supplemental Tables 5 and 6. Survival estimates at 4-year follow-up were 62.1% (95% CI: 60.0%-64.3%) in the VFMR group and 68.1% (95% CI: 65.5%-70.7%) in the AFMR group with no significant difference between the 2 groups (HR: 1.10; 95% CI: 0.99-1.21; *P* = 0.065) (Figure 3). On the other hand, the cumulative incidence of HF hospitalization at 4 years was significantly higher in the VFMR group (46.7%; 95% CI: 44.6%-48.8%) compared to the AFMR group (31%; 95% CI: 28.6%-33.5%; HR: 1.76; 95% CI: 1.57-1.97; *P* < 0.001) (Figure 3). In multivariable analysis, VFMR was an independent predictor of HF hospitalization (HR: 1.57; 95% CI: 1.40-1.77; *P* < 0.0001).

## Discussion

We report the long-term clinical outcomes of patients with moderate and moderate-severe MR with long-term follow-up in contemporary practice. The main findings of the study are as follows: First, clinical event rates were high in this cohort with one-third of patients dying at a median follow-up of 4 years. Second, patients with functional MR had higher unadjusted rates of mortality and HF hospitalization compared to degenerative MR, but these differences were not significant after adjustment for baseline comorbidities and echocardiographic parameters. Third, the cumulative incidence of MV intervention was low. The management of patients with valvular heart disease has historically focused on those with severe symptomatic disease with interventions improving clinical outcomes including survival.^5^ Our study provides insights into this gap with real-world outcome estimates in a large, unselected population. In this cohort of patients, moderate or moderate-severe MR was associated with worst outcomes especially in those with adverse cardiac remodeling, suggesting that this population may represent an appropriate target for future randomized trials evaluating earlier procedural intervention.

Most notable in our findings was the low 4-year survival estimate with one-third of patients dying by 4 years but only 1 in 10 patients having undergone a mitral intervention. Survival estimates were significantly higher in those with degenerative MR compared to functional MR; however, the difference was primarily driven by baseline clinical and echocardiographic characteristics. Among patients with degenerative MR, survival estimates are comparable to previous cohorts of patients with moderate degenerative MR.^11^^,^^12^ Importantly, patients were censored at the time of MV intervention, and our survival estimates reflect clinical outcomes under medical management alone. Hence, the similarity in survival compared to historical studies is expected.

In patients with functional MR, long-term survival in our cohort was higher compared to previous cohorts of functional MR^13^, ^14^, ^15^, ^16^ in part owing to a larger number of atrial FMR patients with relatively higher LVEFs. In a cohort of 1,103 patients with functional MR and reduced ejection fraction, survival estimates were reported to be 48% ± 2% at a median follow-up of 4.1 years.^13^ The difference between these 2 cohorts can be expected as survival is lower in patients with reduced ejection fraction and only 53% of patients in our cohort of functional MR had a LVEF <50%. Another large study utilizing natural language processing included 233,018 patients with moderate MR and reported a 5-year mortality rate of 49%.^14^ This study included a cohort of patients over a 20-year period, and the difference can be driven by the change in goal-directed medical therapy for patients with FMR over the last 10 years. Overall, these findings are encouraging and suggest potential improvement in survival in these patients which correlated with recent evidence supporting the improved survival with medical therapy alone.

As a secondary endpoint, we reported incidence of HF hospitalization as an important clinical outcome on the basis of resource utilization and its association with subsequent mortality.^17^ Our study is the first to report the rate of HF hospitalization in those with moderate/moderate-severe degenerative MR with a reassuring low cumulative incidence of 17.9% at 4-year follow-up. In contrast, the cumulative incidence of HF hospitalization was significantly higher in those with FMR (36.8%), and highest among those with VFMR (46.7%). A recent prespecified analysis from the RESHAPE-HF2 (The Randomized Investigation of the MitraClip Device in Heart Failure: Second Trial in Patients with Clinically Significant Functional Mitral Regurgitation) trial reported a HF hospitalization incidence rate of 44% at 2 years in the medical therapy arm.^8^^,^^18^ While this is higher compared to the overall FMR group in our cohort, it is comparable to the VFMR subgroup. These results likely reflect similar patient characteristics to those in the VFMR subgroup, as the trial included symptomatic patients with moderate MR and reduced LVEF suggesting that real-world event rates are comparable with those observed in this randomized trial population.

Accordingly, the incidence of procedural intervention in patients with moderate or moderate-severe MR remains modest. In our study, the cumulative incidence of MV intervention at 4 years was 11.4%, with significantly higher rates in patients with degenerative MR compared to those with functional MR. This higher rate of intervention is likely explained by the higher rate of MR progression among patients with degenerative MR and a more robust evidence base for surgical intervention in these patients. Given the recent evidence from the RESHAPE-HF2 trial and as data supporting the benefits of earlier intervention continue to emerge, particularly in patients with moderate or moderate-severe MR, a shift toward more frequent interventions in this population is expected especially since outcome of moderate MR is clearly not benign.

### Study limitations

This study has important limitations. First, it is a retrospective analysis involving patients in whom referral for echocardiogram was performed. Thus, patients without indications for echocardiography may represent a lower risk cohort introducing selection bias. Second, echocardiographic assessments were performed as part of routine clinical care in a tertiary referral center rather than in a standardized research protocol or in the general population, and follow-up imaging was not uniformly scheduled. Furthermore, follow-up echocardiographic data were available in only 48% of patients; therefore, conclusions regarding MR progression should be interpreted in this context and may not be generalizable to the full study population. Third, while MR etiology was classified using a structured framework, no core lab read was employed in the present study. However, all TTEs were read by an experienced level 3 trained echocardiologist. Finally, we censored patients at the time of MV intervention to isolate the natural history of moderate MR under medical management, but this approach may underestimate the overall disease burden in patients who ultimately underwent intervention. Additionally, degenerative MR was defined solely by the presence of MV prolapse; other forms of degenerative MR without prolapse may have been classified as functional MR.

## Conclusions

Patients with moderate or moderate-severe MR accrue significant mortality and morbidity over long-term follow-up. In contemporary practice intervention is infrequent in keeping with current guidelines. Cardiac remodeling, especially low LVEF, remains a strong predictor of outcomes in all forms of MR. Further randomized trials are needed to determine whether procedural intervention improves outcomes in these patients and to identify the populations most likely to benefit.

Perspectives **COMPETENCY IN MEDICAL KNOWLEDGE:** Moderate MR is commonly encountered in clinical practice, yet outcomes may vary depending on the underlying etiology. In this large multicenter cohort, patients with functional MR experienced significantly higher risks of HF hospitalization and mortality compared with those with degenerative MR. These findings highlight the importance of considering MR etiology when evaluating patients with moderate MR and may support closer follow-up and optimization of guideline-directed medical therapy in patients with functional MR.

**TRANSLATIONAL OUTLOOK:** Future studies are needed to determine whether intervention in patients with moderate/moderate-severe MR could improve clinical outcomes, particularly among those with functional MR who appear to be at higher risk for adverse events. Identifying the appropriate timing for intervention and the patient populations most likely to benefit will be critical for guiding future therapeutic strategies.

## Funding support and author disclosures

The authors have reported that they have no relationships relevant to the contents of this paper to disclose.
