# Supplementary material for: Natural History and Outcomes of Patients With Moderate and Moderate-Severe Mitral Regurgitation
Source: JACC Adv. 2026 Jul 23;5(8):103057. doi: 10.1016/j.jacadv.2026.103057 (PMC13427544; doi:10.1016/j.jacadv.2026.103057)
Supplement: Supplemental_Material [file mmc1.docx]

| **SUPPLEMENTAL MATERIAL** | | |
| --- | --- | --- |
| **Supplemental material item** | **Description** | **Page** |
| Table 1 | Multivariable cox regression for all-cause mortality | 2 |
| Table 2 | Multivariable Fine-Grey regression for heart failure hospitalization | 3 |
| Table 3 | Multivariable Fine-Grey regression for progression to severe mitral regurgitation | 4 |
| Table 4 | Multivariable Fine-Grey regression for mitral valve intervention | 5 |
| Table 5 | Baseline characteristics of the secondary mitral regurgitation subgroups | 6 |
| Table 6 | Baseline echocardiogram of the secondary mitral regurgitation subgroups | 7 |
|  |  |  |

| **Supplementary Table 1. Multivariable cox regression for all-cause mortality** | | |
| --- | --- | --- |
|  | HR (95% CI) | P value |
| Age | 1.051 (1.046, 1.057) | <0.001 |
| Female | 1.829 (0.746, 0.923) | 0.001 |
| **Functional mitral regurgitation** | **1.085 (0.915, 1.286)** | **0.347** |
| Moderate-severe mitral regurgitation | 2.731 (1.211, 6.161) | 0.016 |
| Body Mass Index | 0.982 (0.973, 0.991) | <0.001 |
| Diabetes Mellitus | 1.238 (1.112, 1.378) | <0.001 |
| Pulmonary Disease | 1.528 (1.331, 1.754) | <0.001 |
| Renal Disease | 1.447 (1.284, 1.632) | <0.001 |
| Coronary Artery Disease | 1.057 (0.946, 1.180) | 0.331 |
| Hypertension | 0.953 (0.835, 1.087) | 0.473 |
| Stroke | 1.258 (1.112, 1.423) | <0.001 |
| History of Congestive Heart Failure | 1.088 (0.973, 1.218) | 0.14 |
| Atrial Fibrillation | 1.036 (0.929, 1.156) | 0.522 |
| Left Ventricular Ejection Fraction | 0.989 (0.985, 0.993) | <0.001 |
| Mitral Annular Calcification | 1.095 (0.988, 1.213) | 0.084 |
| Left Atrial Volume Index | 1.004 (1.002, 1.007) | 0.001 |
| Medial E/e' Ratio | 1.015 (1.009, 1.021) | <0.001 |
| Right Ventricular Systolic Pressure | 1.018 (1.014, 1.022) | <0.001 |
| Moderate or more Tricuspid Regurgitation | 1.026 (0.920, 1.143) | 0.65 |
| Left Ventricular Hypertrophy | 1.242 (1.111, 1.388) | <0.001 |
| Right Ventricular Dilation | 1.044 (0.937, 1.162) | 0.437 |

| **Supplementary Table 2. Multivariable Fine-Grey regression for heart failure hospitalization** | | |
| --- | --- | --- |
|  | HR (95% CI) | P value |
| Age | 1.000 (0.996, 1.004) | 0.851 |
| Female | 1.079 (0.978, 1.192) | 0.13 |
| **Functional Mitral Regurgitation** | **1.029 (0.889, 1.191)** | **0.703** |
| Moderate-Severe Mitral Regurgitation | 1.179 (1.064, 1.307) | 0.002 |
| Body Mass Index | 1.017 (1.009, 1.024) | <0.001 |
| Diabetes Mellitus | 1.194 (1.083, 1.316) | <0.001 |
| Chronic Pulmonary Disease | 1.241 (1.095, 1.407) | <0.001 |
| Renal Disease | 1.176 (1.052, 1.315) | 0.004 |
| Coronary Artery Disease | 1.019 (0.919, 1.129) | 0.726 |
| Hypertension | 1.124 (1.003, 1.259) | 0.045 |
| Stroke | 1.029 (0.909, 1.165) | 0.655 |
| History of Congestive Heart Failure | 1.573 (1.410, 1.755) | <0.001 |
| Atrial Fibrillation | 1.030 (0.931, 1.139) | 0.569 |
| Left Ventricular Ejection Fraction | 0.988 (0.984, 0.991) | <0.001 |
| Left Atrial Volume Index | 1.002 (0.999, 1.004) | 0.173 |
| Mitral Annular Calcification | 1.012 (0.915, 1.120) | 0.813 |
| Medial E/e' Ratio | 1.008 (1.002, 1.013) | 0.006 |
| Left Ventricular Hypertrophy | 1.080 (0.972, 1.199) | 0.154 |
| Right Ventricular Dilation | 1.088 (0.987, 1.200) | 0.091 |
| Right Ventricular Systolic Pressure | 1.004 (1.000, 1.008) | 0.033 |
| Moderate or more Tricuspid Regurgitation | 1.033 (0.933, 1.145) | 0.532 |

| **Supplementary Table 3. Multivariable Fine-Grey regression for progression to severe mitral regurgitation** | | |
| --- | --- | --- |
|  | HR (95% CI) | P value |
| Age | 0.997 (0.991, 1.003) | 0.321 |
| Female | 0.807 (0.692, 0.941) | 0.006 |
| **Functional Mitral Regurgitation** | **0.649 (0.535, 0.788)** | **<0.001** |
| Moderate-Severe Mitral Regurgitation | 2.491 (2.157, 2.877) | <0.001 |
| Body Mass Index | 1.005 (0.992, 1.018) | 0.469 |
| Diabetes Mellitus | 1.144 (0.939, 1.392) | 0.182 |
| Chronic Pulmonary Disease | 1.310 (1.000, 1.717) | 0.05 |
| Renal Disease | 1.223 (0.951, 1.573) | 0.117 |
| Coronary Artery Disease | 0.987 (0.827, 1.179) | 0.889 |
| Hypertension | 0.797 (0.671, 0.947) | 0.01 |
| Stroke | 0.732 (0.555, 0.965) | 0.027 |
| History of Congestive Heart Failure | 1.009 (0.829, 1.228) | 0.929 |
| Atrial Fibrillation | 0.801 (0.669, 0.960) | 0.016 |
| Left Ventricular Ejection Fraction | 1.002 (0.995, 1.008) | 0.637 |
| Left Atrial Volume Index | 1.009 (1.005, 1.013) | <0.001 |
| Mitral Annular Calcification | 1.038 (0.868, 1.241) | 0.686 |
| Medial E/e' Ratio | 1.005 (0.995, 1.015) | 0.366 |
| Left Ventricular Hypertrophy | 1.175 (1.004, 1.376) | 0.045 |
| Right Ventricular Dilation | 1.200 (1.008, 1.427) | 0.04 |
| Moderate or more Tricuspid Regurgitation | 0.854 (0.714, 1.022) | 0.086 |
| Right Ventricular Systolic Pressure | 0.999 (0.992, 1.007) | 0.861 |

| **Supplementary Table 4. Multivariable Fine-Grey regression for mitral valve intervention** | | |
| --- | --- | --- |
|  | HR (95% CI) | P value |
| Age | 0.989 (0.983, 0.996) | <0.001 |
| Female | 0.693 (0.587, 0.819) | <0.001 |
| **Functional Mitral Regurgitation** | **0.454 (0.370, 0.557)** | **<0.001** |
| Moderate-Severe Mitral Regurgitation | 2.910 (2.493, 3.397) | <0.001 |
| Body Mass Index | 1.014 (1.000, 1.029) | 0.049 |
| Diabetes Mellitus | 1.095 (0.856, 1.403) | 0.47 |
| Chronic Pulmonary Disease | 0.879 (0.612, 1.263) | 0.485 |
| Renal Disease | 0.652 (0.450, 0.944) | 0.023 |
| Coronary Artery Disease | 0.912 (0.744, 1.119) | 0.378 |
| Hypertension | 0.637 (0.529, 0.766) | <0.001 |
| Stroke | 0.540 (0.358, 0.813) | 0.003 |
| History of Congestive Heart Failure | 1.062 (0.825, 1.366) | 0.641 |
| Atrial Fibrillation | 0.833 (0.675, 1.027) | 0.086 |
| Left Ventricular Ejection Fraction | 1.035 (1.027, 1.043) | <0.001 |
| Left Atrial Volume Index | 1.013 (1.009, 1.017) | <0.001 |
| Mitral Annular Calcification | 1.075 (0.874, 1.321) | 0.496 |
| Medial E/e' Ratio | 0.991 (0.978, 1.005) | 0.229 |
| Left Ventricular Hypertrophy | 1.214 (1.033, 1.426) | 0.019 |
| Right Ventricular Dilation | 1.029 (0.845, 1.253) | 0.778 |
| Moderate or more Tricuspid Regurgitation | 0.820 (0.666, 1.011) | 0.064 |
| Right Ventricular Systolic Pressure | 0.993 (0.984, 1.002) | 0.128 |

| **Supplementary Table 5. Baseline characteristics of the functional mitral regurgitation subgroups** | | | |
| --- | --- | --- | --- |
|  | AFMR  (N=1828) | VFMR  (N=2678) | p value |
| Age | 75.5 (11.3) | 68.3 (14.1) | < 0.001 |
| Female | 993 (54.3%) | 968 (36.1%) | < 0.001 |
| Body mass index | 29.1 (6.3) | 29.4 (6.3) | 0.108 |
| Body surface area | 1.9 (0.3) | 2.0 (0.3) | < 0.001 |
| Hypertension | 1328 (72.6%) | 1680 (62.7%) | < 0.001 |
| Diabetes mellitus | 485 (26.5%) | 885 (33.0%) | < 0.001 |
| Coronary artery disease | 769 (42.1%) | 1484 (55.4%) | < 0.001 |
| Percutaneous coronary intervention | 105 (5.7%) | 311 (11.6%) | < 0.001 |
| Heart failure | 567 (31.0%) | 1710 (63.9%) | < 0.001 |
| Atrial fibrillation | 1013 (55.4%) | 984 (36.7%) | < 0.001 |
| Stroke | 251 (13.7%) | 318 (11.9%) | 0.065 |
| Lung disease | 203 (11.1%) | 255 (9.5%) | 0.084 |
| Obstructive sleep apnea | 524 (28.7%) | 694 (25.9%) | 0.041 |
| Renal disease | 363 (19.9%) | 445 (16.6%) | 0.005 |
| Non-skin cancer | 435 (23.8%) | 474 (17.7%) | < 0.001 |

AFMR: atrial functional mitral regurgitation, VFMR: ventricular functional mitral regurgitation.

| **Supplementary Table 6. Baseline echocardiogram of the functional mitral regurgitation subgroups** | | | |
| --- | --- | --- | --- |
|  | AFMR  (N=1,828) | VFMR  (N=2,678) | p value |
| MR grade |  |  | < 0.001 |
| Moderate | 1451 (79.4%) | 1894 (70.7%) |  |
| Moderate-severe | 377 (20.6%) | 784 (29.3%) |  |
| Effective regurgitant orifice area | 0.24 (0.06) | 0.26 (0.07) | < 0.001 |
| Regurgitant volume | 43.1 (9.3) | 41.4 (9.6) | < 0.001 |
| Mitral annular calcification | 678 (37.1%) | 668 (24.9%) | < 0.001 |
| Ejection fraction | 60.0 (5.4) | 30.3 (9.7) | < 0.001 |
| Ejection fraction ≤50% | 0 (0.0%) | 2678 (100.0%) | < 0.001 |
| LV end-systolic diameter | 33.2 (5.2) | 53.5 (8.9) | < 0.001 |
| LV end-diastolic diameter | 50.3 (6.0) | 62.9 (7.9) | < 0.001 |
| E wave | 1.0 (0.3) | 0.9 (0.3) | < 0.001 |
| Medial E/é ratio | 17.0 (7.6) | 21.1 (9.8) | < 0.001 |
| Left ventricular mass index | 109.7 (29.9) | 142.5 (36.1) | < 0.001 |
| Left atrial volume index | 57.5 (18.9) | 53.6 (16.7) | < 0.001 |
| TR velocity | 2.93 (0.47) | 2.95 (0.48) | 0.155 |
| Right ventricular systolic pressure | 44.0 (13.4) | 45.5 (13.6) | < 0.001 |
| Tricuspid regurgitation |  |  | < 0.001 |
| None/Trivial | 295 (16.4%) | 553 (21.2%) |  |
| Mild | 559 (31.1%) | 903 (34.7%) |  |
| Moderate | 666 (37.0%) | 838 (32.2%) |  |
| Severe | 280 (15.6%) | 312 (12.0%) |  |
| Right ventricular dilation | 712 (39.1%) | 1338 (50.2%) | < 0.001 |
| Right ventricular dysfunction | 479 (26.2%) | 1576 (58.8%) | < 0.001 |

AFMR: atrial functional mitral regurgitation, LV: left ventricle, MR: mitral regurgitation, TR: tricuspid regurgitation, VFMR: ventricular functional mitral regurgitation
